# A pro-angiogenic wound dressing embedded with natural spider silk protein

**DOI:** 10.1093/rb/rbaf078

**Published:** 2025-07-29

**Authors:** Sai Yan, Zhou Zhang, Yuheng Song, Juan Zhao, Hanrui Wang, Xiang Fei, Ran Cao, Meifang Zhu

**Affiliations:** State Key Laboratory of Advanced Fiber Materials, College of Materials Science and Engineering, Donghua University, Shanghai 201620, China; State Key Laboratory of Advanced Fiber Materials, College of Materials Science and Engineering, Donghua University, Shanghai 201620, China; State Key Laboratory of Advanced Fiber Materials, College of Materials Science and Engineering, Donghua University, Shanghai 201620, China; Hainan Spider King Biotechnology Co, Ltd, Haikou 570125, China; Hainan Spider King Biotechnology Co, Ltd, Haikou 570125, China; State Key Laboratory of Advanced Fiber Materials, College of Materials Science and Engineering, Donghua University, Shanghai 201620, China; State Key Laboratory of Advanced Fiber Materials, College of Materials Science and Engineering, Donghua University, Shanghai 201620, China; Shanghai Engineering Research Center of Nano-Biomaterials and Regenerative Medicine, Donghua University, Shanghai 201620, China; State Key Laboratory of Advanced Fiber Materials, College of Materials Science and Engineering, Donghua University, Shanghai 201620, China

**Keywords:** natural spider silk, nanofiber, pro-angiogenic, wound healing

## Abstract

Angiogenesis plays a pivotal role in the wound healing process by supplying essential nutrients and oxygen to regenerating tissues thereby supporting tissue remodeling. Promoting the formation of new blood vessels is, therefore, a critical therapeutic strategy, particularly for ischemic and chronic wounds, where impaired blood supply often leads to delayed or incomplete healing. However, the development of effective pro-angiogenic biomaterials remains a challenge. In this work, by incorporating natural spider silk proteins (SSP) with poly(L-lactic acid) (PLLA) nanofiber via electrospinning, we developed a pro-angiogenic wound dressing. The incorporation of SSP led to a reduction in fiber diameter and the formation of a hierarchical structure that mimics the native extracellular matrix. Moreover, the combined effects of these biophysical and SSP-derived biochemical cues synergistically enhanced vascular regeneration, resulting in significant improvements in three key angiogenic parameters compared to pure PLLA controls: a 16.3% increase in blood vessel count, a 118.6% increase in vascular branching and a 32.8% increase in total vessel length. *In vivo* wound healing experiments showed a 29% improvement in the wound healing rate compared to the control group. This dual-mechanism strategy, synergizing structural biomimicry with bioactive cues, establishes a multifunctional platform to address complex wound healing challenges, particularly in ischemic and chronic wounds.

## Graphical abstract

**Figure:**
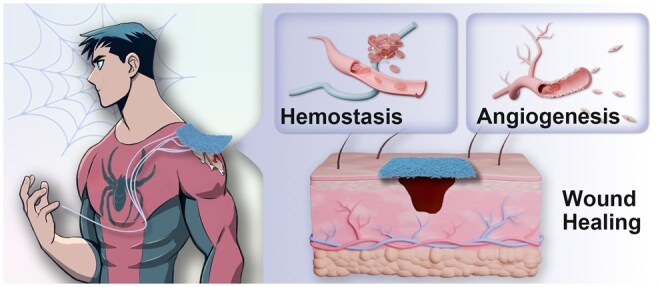

## Introduction

Skin, the body’s largest organ, plays a crucial role in defending against external microbial invasion and regulating endocrine balance [1]. However, hundreds of millions of people suffer from wounds each year due to illness, surgery or accidents, which poses significant challenges to both patients and healthcare systems [2]. Wound healing is a complex series of cascading reactions involving intricate interactions between cells, growth factors and the extracellular matrix [3, 4]. By addressing these cascades during the healing process, targeted interventions can be employed to prevent wound deterioration and promote healing [5, 6]. Angiogenesis is a critical step in these cascaded processes. Enhanced angiogenesis promotes the delivery of nutrients and oxygen to the wound site, accompanied by minimal fibrosis, thereby improving wound healing. In recent years, strategies such as the use of recombinant growth factors that promote angiogenesis and/or nucleotides encoding angiogenic factors have been explored to enhance vascular regeneration. However, these treatments are costly, challenging to administer effectively and have limited clinical potential [7]. Strategies involving the loading or grafting of growth factors—such as vascular endothelial growth factor (VEGF), basic fibroblast growth factor (bFGF) and transforming growth factor (TGF)—have been widely employed in recent years. Nevertheless, rapid degradation and functional deactivation of these factors remain persistent challenges [8, 9]. Nanotechnology offers new perspectives for wound vascularization through nanoparticles (e.g. gold/zinc oxide/ceria/bioactive glass) and carbon-based materials (e.g. graphene). Nevertheless, concerns regarding dispersion stability and biocompatibility require resolution [10, 11]. To overcome these challenges, the development of safe and effective pro-angiogenic wound dressings is crucial.

Electrospun nanofibers have emerged as promising wound dressings due to their unique nanoscale properties, including high surface area and porosity. Additionally, their extracellular matrix (ECM)-like structure provides a complex, spatiotemporal platform for biochemical and biophysical cues, which play a critical role in guiding cellular activities during the healing process, including angiogenesis. These properties have led to their widespread use in developing advanced wound dressings [12–15]. Among the polymers employed in electrospinning, natural polymers such as gelatin, collagen and chitosan [16–18], typically feature abundant functional groups and exhibit excellent bioactivity. However, they often suffer from poor mechanical properties and stability. In contrast, synthetic polymers like polylactic acid (PLA), polycaprolactone (PCL) and polyvinylpyrrolidone (PVP) [19–21], offer high controllability but lack sufficient bioactivity and cellular interaction capabilities [3, 22, 23]. To this end, the synergistic use of both natural and synthetic polymers has emerged as an effective strategy for designing bioactive, customizable and adjustable composite wound dressings.

Among diverse electrospinning materials, silk-based biomaterials hold unique status. Natural silks—produced by arthropods (arachnids, insects, myriapods)—exhibit exceptional biocompatibility and distinctive bioactivity, enabling broad regenerative applications [24]. Crucially, spider silk diverges from silkworm silk in amino acid sequences, secondary structures and tertiary configurations, conferring differentiated mechanical properties and biological functions [25, 26]. For example, the well-known one is the higher β-sheet content forms ‘nano-fishing-net configurations’ that impart extraordinary strength and toughness [27]. Moreover, compared with silkworm silk, the biochemical and biophysical properties of spider silk proteins (SSP) facilitate interactions between cells and the ECM, providing cues that rapidly recruit and stimulate cells. These interactions positively influence cellular behavior and tissue repair, effectively mimicking and supporting tissue growth and regeneration [28–30]. For example, Thomas Scheibel and colleagues developed 3D-printed scaffolds for soft tissue repair by reinforcing collagen with the recombinantly produced SSP eADF4(C16). Compared to scaffolds made with unmodified eADF4(C16) variants, the reinforced scaffolds showed improved biocompatibility, biodegradability and bioactivity [31]. Baoyong et al. reported that recombinant SSP pNSR-16 and pNSR-32 accelerated the healing of deep second-degree burn wounds, observing increased expression and secretion of growth factors like bFGF and hydroxyproline [32]. However, research on spider silk in the field of regenerative repair has primarily focused on recombinantly produced spider silk, and the study of natural spider silk for wound healing remains largely unexplored.

Here, we developed a new wound dressing with enhanced angiogenic and wound-healing properties by electrospinning natural SSP into polymer nanofibers. Specifically, we purified SSP from the naturally sourced *Chilobrachys guangxiensis* spider silk and combined them with poly(L-lactic acid) (PLLA) for electrospinning. The as prepared nanofiber scaffold (PLLA/SSP) demonstrated excellent cell compatibility and blood compatibility. Furthermore, the bioactivity of PLLA/SSP was investigated, including its hemostatic and angiogenic properties. Finally, PLLA/SSP showed remarkable therapeutic effects in a rat full-thickness skin defect model, promoting angiogenesis, re-epithelialization and granulation tissue formation.

## Materials and methods

### Materials

Poly-L-lactic acid (PLLA) was purchased from Jinan Daigang Biomaterial, Jinan, China. 1,1,1,3,3,3-Hexafluoro-2-propanol (HFIP) was purchased from Bidepharm, Shanghai, China. *Chilobrachys guangxiensis* spider silk was provided by Hainan Spider King Biotechnology, Haikou, China. Phosphate buffered saline (PBS) was purchased from Macklin, Shanghai, China.

### Purification of SSP

Spider silk produced by *Chilobrachys guangxiensis* under simulated natural living conditions was carefully harvested by hand. The harvested spider silk was initially separated to remove food debris and excrement. It was then stirred in deionized water overnight to further eliminate dust and other impurities. After washing, the spider silk was dissolved in HFIP at 50°C to achieve an SSP solution (∼0.6 mg/mL). This solution was subsequently concentrated at 65°C to obtain the SSP concentrated solution (∼6 mg/mL).

### Fabrication of electrospun membranes

One gram of PLLA was dissolved in 5 mL HFIP and stirred sufficiently to obtain the spinning precursor solution. Electrospinning was conducted for 2 h at 15 kV voltage and a flow rate of 1.0 mL/h to produce pure PLLA nanofiber membranes. Similarly, 1 g of PLLA was dissolved in 5 mL SSP solution to obtain low SSP content electrospun membranes (PLLA/SSP-L), and in 5 mL SSP concentrated solution to obtain high SSP content electrospun membranes (PLLA/SSP-H).

### Characterization of the electrospun membranes

The scanning electron microscopy (SEM) images of the samples were acquired using a Hitachi SU8010. The micro-morphology and roughness of the samples were characterized using a Dimension FastScan/Icon model atomic force microscope (AFM). The Fourier transform infrared (FTIR) tests of the SSP and PLLA/SSP were obtained using a Nergers Nicolet 6700. Elemental distribution was analyzed by a Bruker energy spectrometer (GeminiSEM 560). Water contact angle (WCA) analysis was performed on the surface of the electrospinning membrane using a contact angle meter (Theta Flex, Biolin Instrument). The diameters of electrospun nanofibers were estimated using image processing software ImageJ software (National Institute of Health, USA).

### *In vitro* cytocompatibility

The *in vitro* cytocompatibility of electrospun membranes was evaluated using the Cell Counting Kit-8 (CCK-8) assay with mouse fibroblast cells (L929) as the model. Electrospun membranes were cut into 1 cm diameter discs and placed in 24-well plates, with each well supplemented a culture medium composed of 89% Dulbecco’s Modified Eagle Medium (DMEM), 10% fetal bovine serum and 1% antibiotics (penicillin and streptomycin). Each well was seeded with 15 000 cells and cultured at 37°C with 5% CO_2_ for 1, 3 and 5 days. After each incubation period, the culture medium was removed, and CCK-8 reagent was added. Following a 1-h incubation protected from light, the supernatant absorbance was measured at 450 nm using a microplate reader. A control group containing cells and culture medium containing no sample was used as a blank. Calculate the relative cellular activity (RCA) using the formula: RCA = *A*_Sample_/*A*_Blank_ × 100%, where *A*_Sample_ is the sample absorbance, and *A*_Blank_ is the control absorbance.

The viability and morphology of L929 cells in contact with electrospun membranes were assessed using live/dead staining. Briefly, after 5 days of culture, the culture medium was removed, and a fluorescent Calcein AM viability assay was added to the sample, followed by incubation at 37°C for 20 min. Cell observation was conducted using a fluorescence microscope (Olympus BX53).

Furthermore, L929 cells on electrospun membranes cultured for 3 days were fixed, consulting the procedure similar to that described in *in vitro* cytocompatibility section for fixing blood cells on sample surfaces. Subsequently, the morphologies of L929 cells on the electrospun membranes were observed using SEM.

### *In vitro* hemocompatibility and PT and APTT test

The hemolysis rate of the material is measured in the following way. Sodium citrate-anticoagulated rabbit blood (ARB) at 3000 rpm for 5 min to pellet red blood cells (RBCs) in a centrifuge tube. Remove the supernatant, resuspend in PBS and repeat centrifugation twice to obtain the RBC pellet. Mix 1 mL of the RBC pellet with 19 mL of PBS to prepare an RBC suspension. Incubate 1 mL of the RBC suspension with 10 μg of electrospun sample at 37°C for 2 h. After centrifuging at 3000 rpm for 5 min, measure supernatant absorbance at 540 nm. Use 50 μL of PBS and 50 μL of 0.1% Triton X-100 as negative and positive controls, respectively. Calculate the hemolysis rate (HR) using the formula: HR = (AS − AN)/(AP − AN) × 100%, where AS is the sample absorbance, AN is the negative control absorbance and AP is the positive control absorbance.

The clotting properties of electrospun membranes were characterized using activated partial thromboplastin time (APTT) and prothrombin time (PT). Sodium citrate-anticoagulated rabbit plasma (ARP), APTT reagent, PT reagent and a 0.2 mol/L CaCl_2_ solution were separately placed in test tubes and incubated at 37°C. 140 µL of ARP was mixed with the electrospun sample, followed by addition of 70 µL of APTT reagent and 140 µL of CaCl_2_ solution. Timing started instantly when the reagents are mixed and stopped when plasma ceased to flow, repeated in triplicate to obtain APTT. Similarly, 140 µL of ARP was mixed with the electrospun sample, followed by addition of 70 µL of PT reagent, timing to obtain PT.

In addition, the hemocompatibility and coagulation properties of the materials were further evaluated by blood cells fixation experiments. ARB was mixed with the electrospun membrane sample and incubated for 30 min. Remove the electrospun membrane samples and wash off excess blood carefully with PBS. Fix the samples in 4% glutaraldehyde solution at 4°C for 1 h. Sequentially immerse them in 30%, 50%, 70%, 90% and two rounds of 100% ethanol for 10 min each, followed by freeze-drying. Finally, assess the hemostatic performance and blood cell compatibility using SEM.

### *In vitro* tube formation assay

To evaluate the effects of PLLA/SSP on angiogenesis, HUVECs were used as a model cell in tube formation assay. 100 μL of Matrigel (Corning, thawed at 4°C overnight) was added to pre-cooling 96-well plates and incubated in 37°C for 30 min. Then, 10 000 HUVECs were seeded into each well with 100 μL of DMEM containing the extract of the electrospun membranes and cultured at 37°C with 5% CO_2_ for 2 h. Images of tube formation were recorded by a microscope (Olympus BX53) and analyzed using ImageJ.

### Animal model healing and histopathology analysis

Animal studies in this study were conducted in strict accordance with the Donghua University Laboratory Animal Ethics Review Committee (DHUEC-MOST-2021-06). Full-thickness wound healing experiments were carried out according to a previous report with minor revisions. The backs of male Sprague-Dawley (SD) rats were shaved under anesthesia and six 10 mm-diameter full-thickness skin defects were incised. Each wound was treated with an electrospun membrane sample, and wounds without sample treatment were used as a blank control. Images of wound sites were taken after 3, 7 and 14 days of treatment, and ImageJ software was used to analyze the changes in wound area. Wound closure rate (WCR) is calculated using the following formula: WCR= (*A*0 − *A*N)/A0 × 100%, where *A*0 refers to the initial wound area and *A*n is the area of the wound after sample treatment. In addition, skin tissue from the wound site was collected and soaked in a 4% paraformaldehyde solution, subsequently sliced and subjected to Hematoxylin-eosin (H&E) and Masson’s trichrome staining and image capture. The histopathology analysis process is performed in the CaseViewer software.

### Static analysis

Statistical analyses were performed using SPSS (SPSS, Inc., Chicago, IL, USA). A paired Student’s t-test was used for statistical analysis in this study. One-way ANOVA and Tukey’s multiple comparisons test with more than two groups was used. All assays were repeated in at least three independent experiments. The ‘ns’ presents *P* > 0.05, representing no significant difference and comparisons with *P < 0.05, **P < 0.01, ***P < 0.001 were considered statistically significant.

## Results and discussion

### Characterization of electrospun nanofiber scaffolds

**Figure 1.**
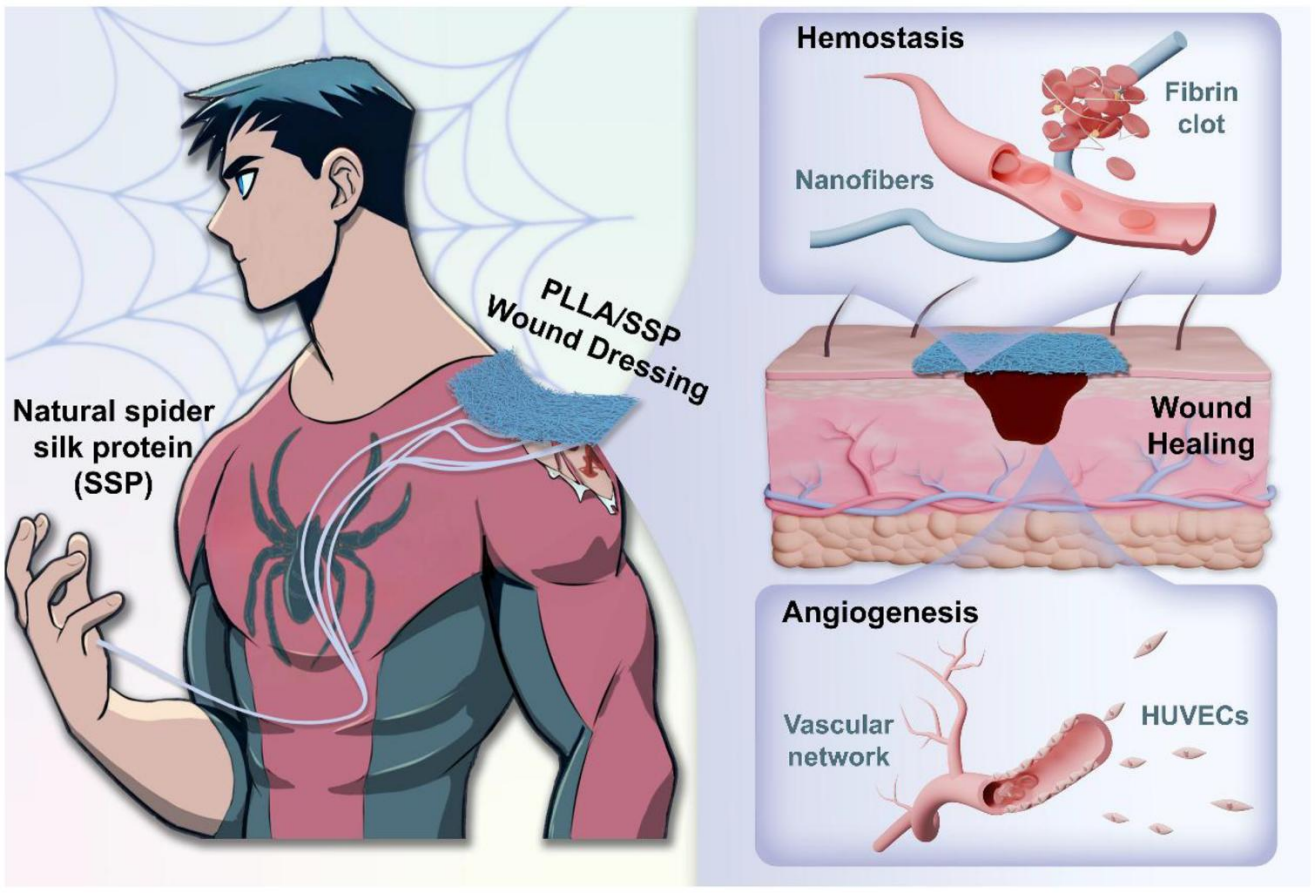
Schematic illustration of the electrospun PLLA/SSP wound dressing and its wound regenerative properties related to hemostasis and angiogenesis.

**Figure 2.**
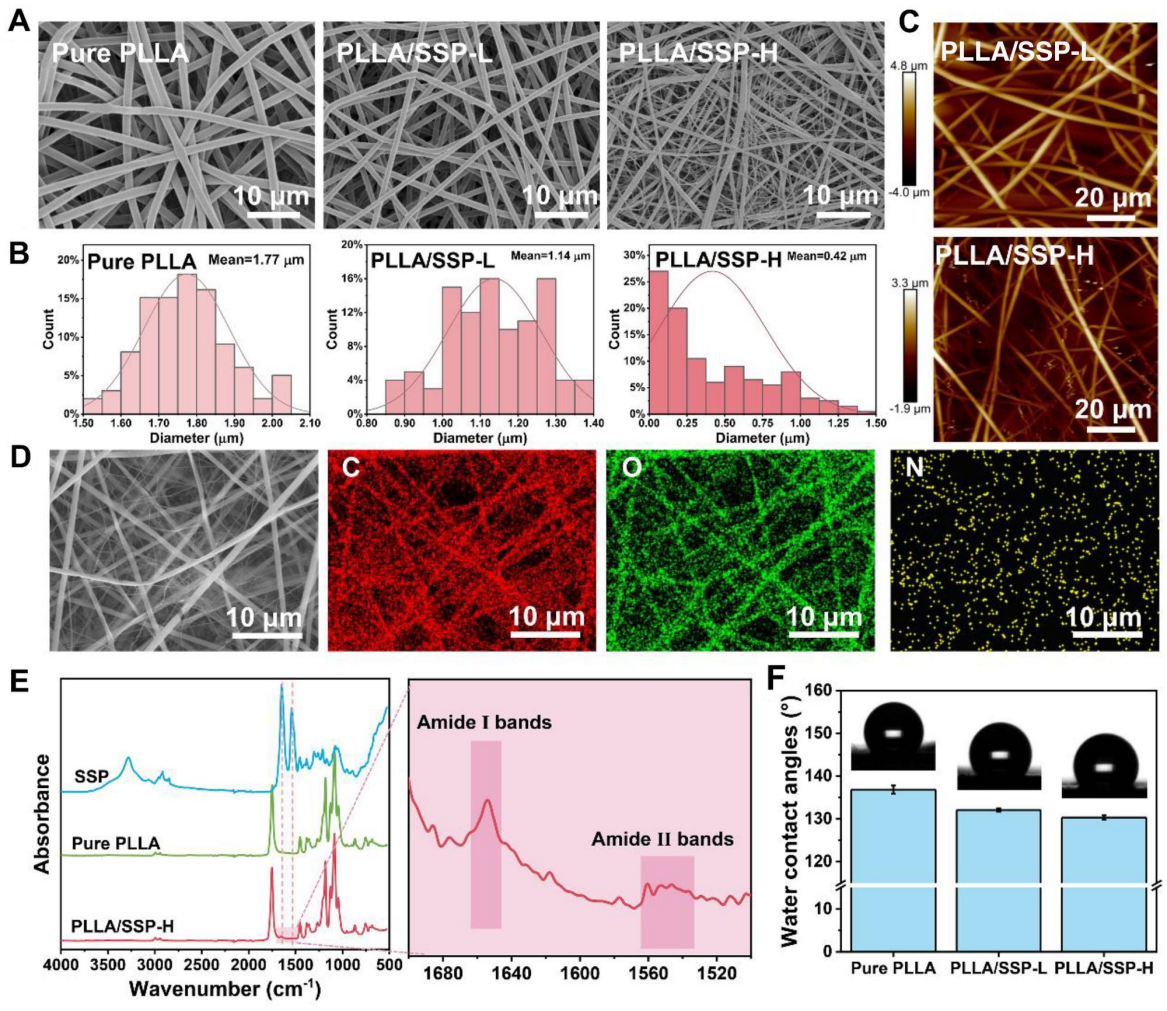
Characterizations of pure PLLA, PLLA/SSP-L and PLLA/SSP-H nanofiber membranes. (**A**) The scanning electron microscopy (SEM) images and (**B**) diameter distribution of electrospun nanofibers. (**C**) The atomic force microscope (AFM) images of PLLA/SSP-L and PLLA/SSP-H. (**D**) The energy dispersive spectrometer (EDS) of PLLA/SSP-H: SEM image, C element, O element and N element. (**E**) The Fourier transform infrared (FTIR) spectra of SSP, pure PLLA and PLLA/SSP-H. (**F**) Water contact angles of nanofiber membranes.

The electrospun PLLA/SSP scaffold is a PLLA-based wound dressing embedded with natural SSP, designed to promote rapid hemostasis, enhance angiogenesis and facilitate the restoration of normal skin structure and function in wounds (Figure 1). Prior to the preparation of SSP-doped electrostatic spinning membranes, purified SSP was characterized using scanning electron microscopy (SEM) and gel permeation chromatography (GPC). As shown in Supplementary Figure S1, the purified SSP consists of a continuous, loosely connected network with spherical ends, with no visible impurities. GPC analysis revealed a molecular weight (Mw) of ∼127 155 g/mol with relatively narrow molecular weight distribution (Supplementary Table S1 and Supplementary Figure S2). Subsequently, the electrospun membranes were prepared and the SEM images of pure PLLA, PLLA/SSP-L and PLLA/SSP-H were depicted in Figure 2A. As the concentration of SSP increases, there is a gradual increase in fibers with smaller diameters, particularly for PLLA/SSP-H (Figure 2B). The average diameter of PLLA is approximately 1.77 μm, while the average diameters of PLLA/SSP-L and PLLA/SSP-H have decreased to 1.14 μm and 417 nm, respectively. This structural evolution enables PLLA/SSP-H to develop a hierarchically graded fiber architecture with two distinct characteristic dimensions: large-scale micron fibers and nanoscale fibers. This phenomenon may be attributed to the optimization of spinnability by the charges of amino acids in SSP in the solution state. The average roughness (Ra) obtained from atomic force microscopy (AFM) measurements further reflects this morphological change (Figure 2C). The Ra reached 1116 nm and 590 nm for PLLA/SSP-L and PLLA/SSP-H, respectively. These findings demonstrate that SSP incorporation effectively modulates fiber diameter and surface roughness, creating a hierarchical micron-to-nanoscale biomimetic microenvironment that closely emulates native extracellular matrix architecture. This structural hierarchy establishes a suitable biophysical foundation for subsequent cellular behavior regulation and tissue regenerative processes through coordinated mechanobiological activation.

The element mapping images of PLLA/SSP-H showed that elements of C, O and N were regularly distributed. Despite the relatively low content of elemental N, it shows a uniform distribution of SSP in the electrospun membrane (Figure 2D). The rich porosity of the nanofiber membrane provides excellent breathability. As a result, the water vapor permeability of PLLA/SSP-H reaches 2231 g·m^−2^·d^−1^ (Supplementary Figure S3). Fourier transform infrared (FTIR) demonstrates the successful doping of SSP in electrospun PLLA fibers. Due to the relatively low content of SSP compared to the polymer, the FTIR spectra of electrospun PLLA/SSP and PLLA are similar but the difference still exists. The FTIR spectrum of PLLA/SSP-H shows peaks at 1652 cm^−1^ and 1550 cm^−1^, which correspond to the amide I and amide II bands of SSP (Figure 2E) [33, 34]. Additionally, Figure 2F shows the water contact angle of the electrospun membrane. With the addition of SSP, the nanofiber membrane’s strong hydrophobicity is reduced, which is beneficial for cell adhesion.

### *In vitro* cytocompatibility and hemocompatibility

**Figure 3.**
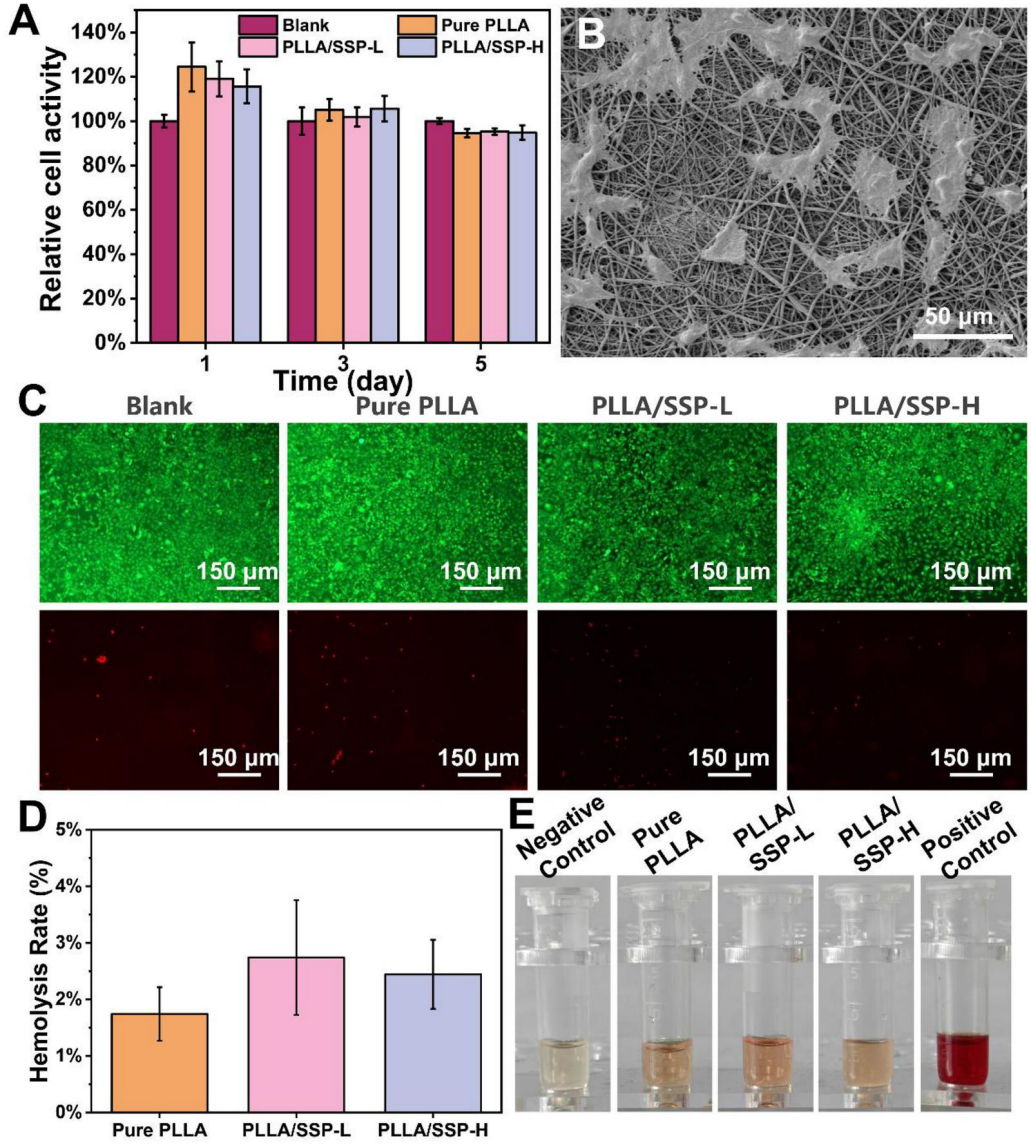
Cytocompatibility and hemocompatibility of nanofiber membranes. (**A**) Relative cell viability of L929 cells on blank, pure PLLA, PLLA/SSP-L and PLLA/SSP-H membranes. (**B**) SEM image of L929 cells adhered to PLLA/SSP-H. (**C**) Live/dead fluorescence microscope images of L929 cells incubated on the nanofiber membranes for 5 days. (**D**) Hemolysis assay results for different nanofiber membranes. (**E**) Images of the extent of hemolysis.

PLLA/SSP wound dressing, as a material in direct contact with the wound site, must exhibit good biocompatibility. Therefore, the biocompatibility of the PLLA/SSP was assessed through CCK-8 and live/dead staining using L929 cells. Based on absorbance measurements, all groups of cells displayed a clear proliferation trend (Supplementary Figure S4). Relative cellular viability of the electrospun nanofiber scaffolds, calculated as shown in Figure 3A, was slightly higher compared to the control group on Day 1 and 3. The decrease in relative cellular viability on Day 5 may be attributed to limited nutrients and space. Overall, the relative cell viability at all time points and in all groups exceeded 90%, indicating that the materials are low cytotoxicity. As shown in Figure 3B and Supplementary Figure S5, SEM confirmed that the cell morphology on the electrospun membrane surface remained intact. Notably, the PLLA/SSP-H surface, exhibited enhanced cell adhesion, increased cell density and the extension of more filopodia, mechanistically attributed to its ECM-mimetic hierarchical architecture that delivers synergistic biophysical cues. Additionally, live/dead staining was used to assess cell-sample interactions at Day 5 (Figure 3C). It was observed that the L929 cells in all groups generally showed great cell viability and cell morphology (green area). The above results confirm the excellent cell growth, adhesion and proliferation of PLLA/SSP, highlighting its outstanding potential as a bioactive wound dressing.

Hemocompatibility is a fundamental requirement for wound dressings, especially those that are in direct contact with blood or tissue fluids. The hemocompatibility of the electrospun membranes was characterized by hemolysis, as shown in Figure 3D and E. All samples had hemolysis rates below 5% (the highest level of the acceptable hemolysis ratio [35, 36]), and pictures of hemoglobin released from red blood cells also showed the low hemolysis activity of the electrospun membrane. These indicate that the prepared electrospun membranes have high hemocompatibility.

### Hemostatic performance

**Figure 4.**
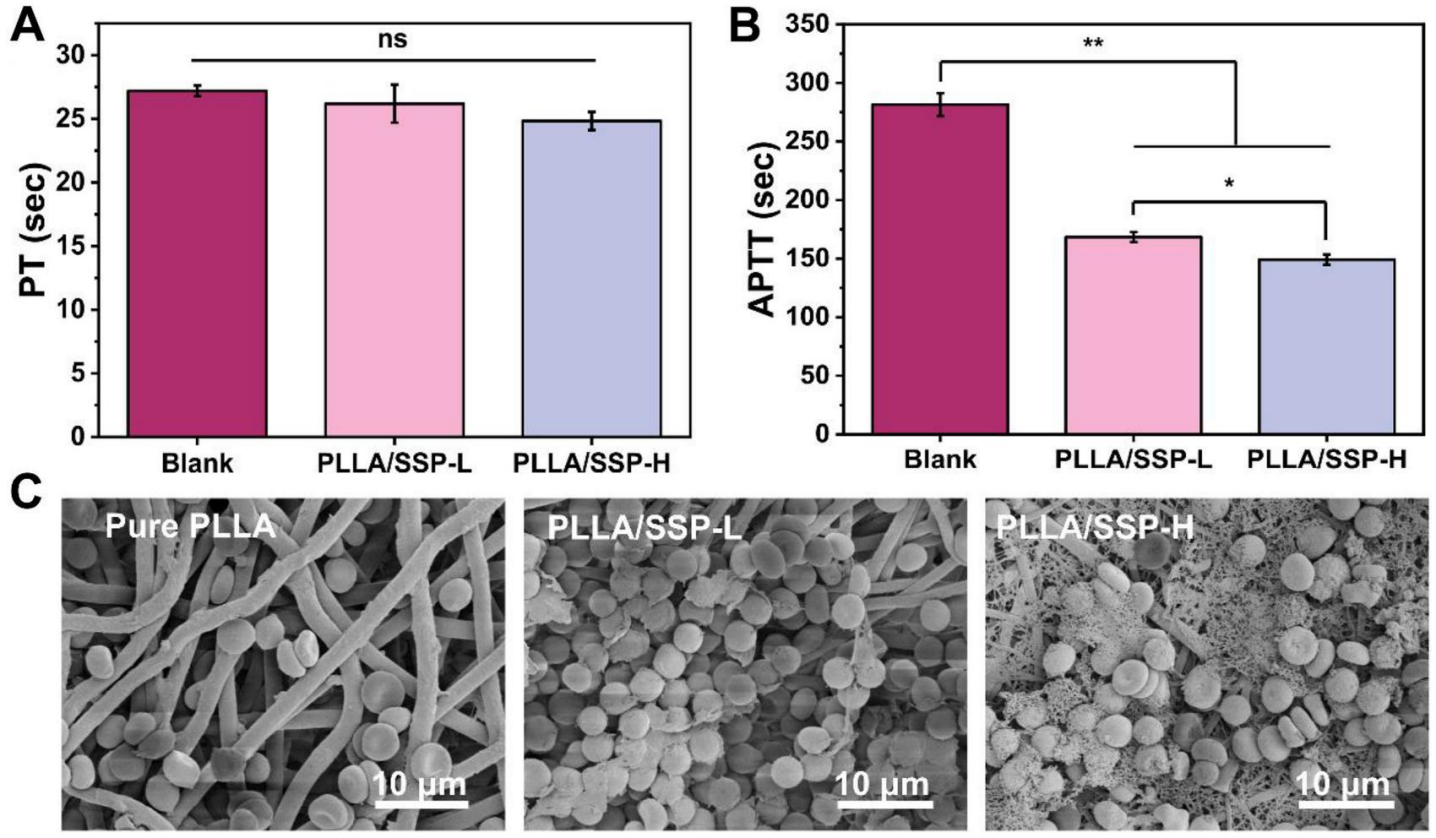
Hemostatic properties of nanofiber membranes. PT (**A**) and APTT (**B**) of blank, PLLA/SSP-L and PLLA/SSP-H. (**C**) SEM images of RBC after different treatments. *, ** denote statistically significant differences between different groups with *P* < 0.05 and *P* < 0.01, respectively.

Hemostasis is a crucial step in the initial stage of wound healing, establishing a temporary barrier to stop bleeding, protect the tissue and prevent potential infection [37, 38]. Consequently, advanced wound dressings should possess the ability to accelerate coagulation. The coagulation properties of the electrospun membranes were assessed using prothrombin time (PT) and activated partial thromboplastin time (APTT) (Figure 4A and B). Compared to the blank group, both PLLA/SSP variants exhibited reduced clotting times, indicating their capacity to enhance both extrinsic and intrinsic coagulation pathways. The effect on the intrinsic pathway was particularly pronounced, with PLLA/SSP-L and PLLA/SSP-H shortening APTT by 40.2% and 47.0% compared to the blank group, respectively. Noteworthily, plasma from the pure PLLA group did not coagulate in either the PT or APTT tests, highlighting the crucial role of SSP modification in enhancing coagulation with PLLA. The zeta potentials of SSP and PLLA/SSP-H were measured at −40.06 ± 1.39 mV and −40.17 ± 2.51 mV, respectively (Supplementary Figure S6). This negative surface charge profile further corroborates the intrinsic coagulation activity observed in PLLA/SSP-H. Morphological examination of red blood cells (RBC) on the surface of electrospun membranes revealed that no cell breakage or morphological changes were observed. Furthermore, it is important to note that the doping of SSP promoted blood cell aggregation and activation. Moreover, the surface of electrospun PLLA/SSP membranes exhibited abundant fibrin formation, indicating the excellent potential of PLLA/SSP in enhancing blood coagulation (Figure 4C). All the above suggest that the functionalization of SSP increases the scaffold’s potential as an embolic hemostatic agent. This phenomenon may be primarily attributed to the synergistic contributions of: (i) the hierarchical architecture of PLLA/SSP-H facilitating physical adsorption of blood components [39], and (ii) activation of coagulation factor XII by its negatively charged surface [40, 41].

### Angiogenic properties

**Figure 5.**
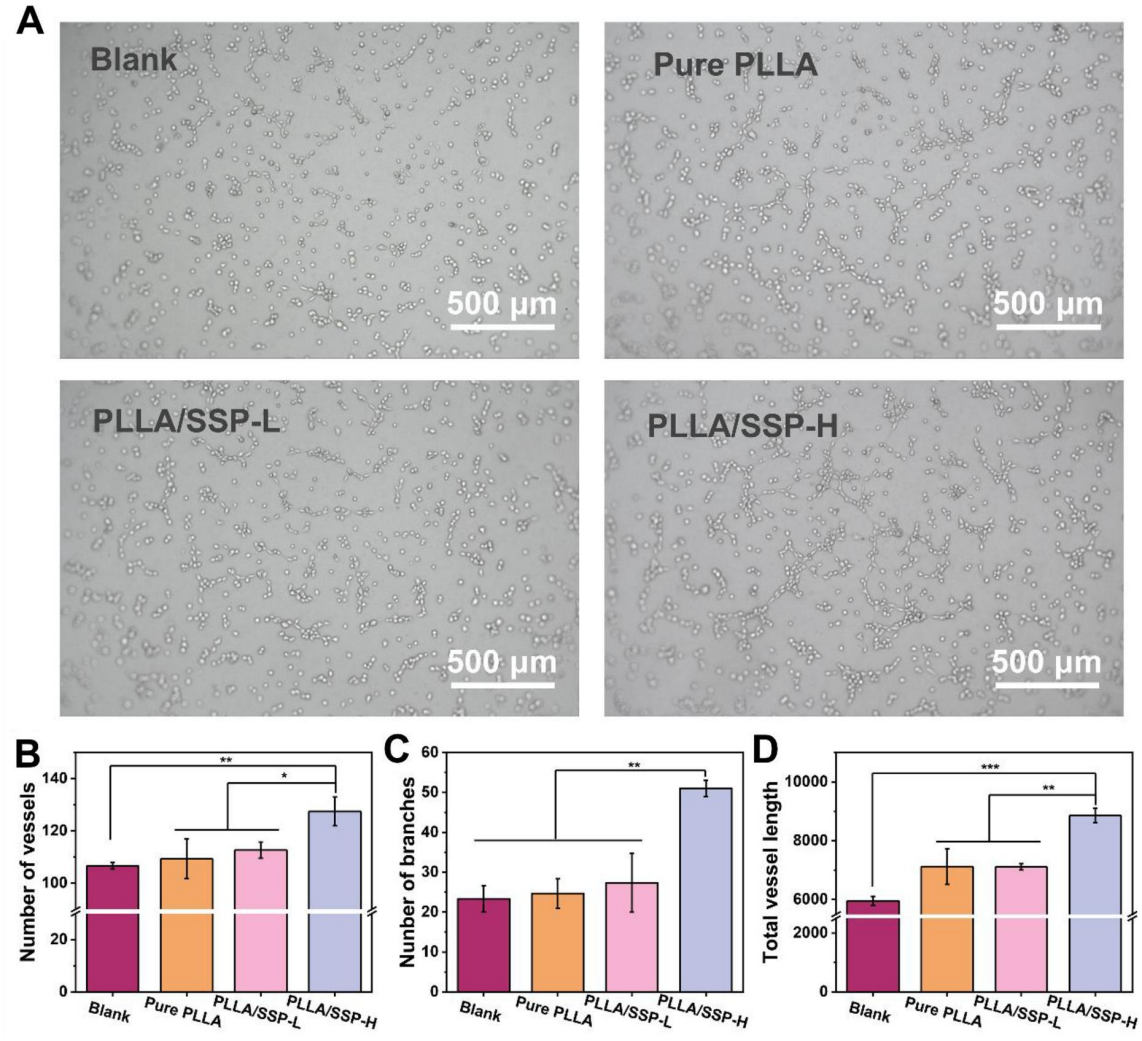
Angiogenic properties of nanofiber membranes. (**A**) Tube formation images of HUVECs with different treatments. Quantitative analysis of number of (**B**) vessels, (**C**) branches and (**D**) total vessel length. *, ** and *** denote statistically significant differences between different groups with *P* < 0.05, *P* < 0.01 and *P* < 0.001, respectively.

Adequate angiogenesis is crucial for wound healing, as neovascularization enables dynamic regulation of cell-microenvironment interactions. Angiogenesis involves endothelial cells forming tubular structures that extend, branch and establish interconnected networks [5, 42]. To assess the angiogenic potential of PLLA/SSP, an *in vitro* tube formation assay was performed using Human Umbilical Vein Endothelial Cells (HUVECs). As shown in Figure 5A, HUVECs in all groups formed capillary-like structures after 2 h of co-culture. However, HUVECs cultured with different membrane extracts exhibited distinct self-assembly behaviors. The number of vessels and total vessel length were significantly greater in the PLLA/SSP-H group compared to both the Blank and pure PLLA groups (Figure 5B–D). Specifically, the number of blood vessels in the PLLA/SSP-H group was 1.2 times higher than in the blank group (Figure 5B). The number of vascular branches increased by 118.6% in the PLLA/SSP-H group compared to the blank group, and by 106.7% compared to the pure PLLA group (Figure 5C). Regarding total vascular length, the PLLA/SSP-H group showed a 32.8% increase compared to the blank group and a 19.6% increase compared to the pure PLLA group (Figure 5D). These results suggest that PLLA/SSP-H has the potential to enhance vascular regeneration and the development of highly mature vascular networks.

### *In vivo* wound healing

**Figure 6.**
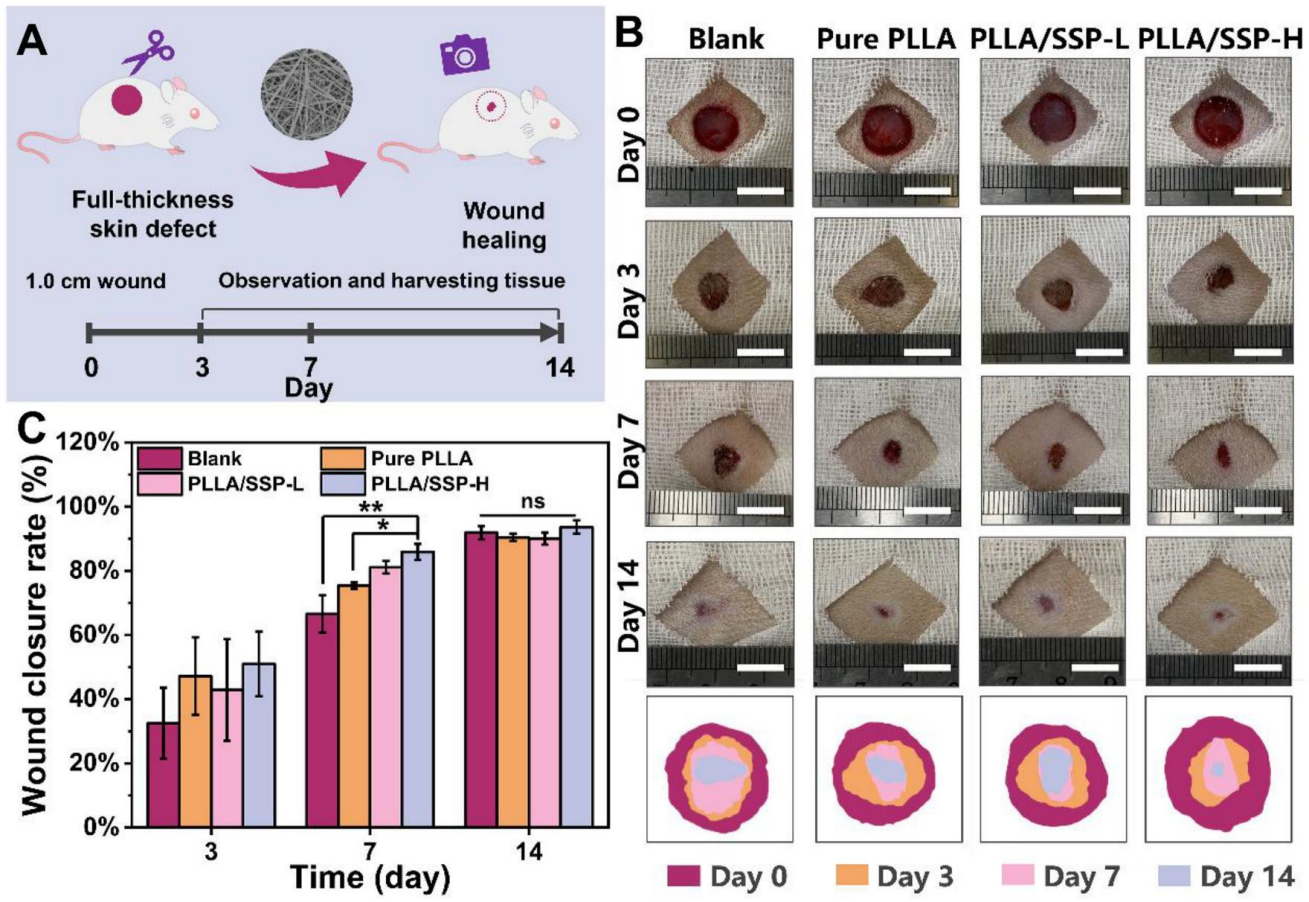
Full-thickness wound healing performance. (**A**) Illustrations of wound formation and treatment. (**B**) Representative images and of wounds and traces of wound closure with varied treatments from Day 0 to Day 14 (scale bar = 1 cm). (**C**) Wound closure rate with different treatments at set time points. *, ** denote statistically significant differences between different groups with *P* < 0.05 and *P* < 0.01, respectively. The ‘ns’ denotes *P* > 0.05, representing no significant difference.

PLLA/SSP exhibits hemostatic, breathable and angiogenic properties, all of which hold promise for wound closure applications. Therefore, full-thickness wounds of 1.0 cm were created on the dorsal side of rats to construct a wound healing model. The injured sites were carefully monitored over a two-week period, with photographs taken on Day 3, 7 and 14, followed by tissue collection and staining (Figure 6A). Digital photographs of the wounds showed that rats treated with PLLA/SSP exhibited significantly faster wound closure compared to the other groups, with all defects nearly fully healed by Day 14 (Figure 6B). Statistical analysis of wound closure (Figure 6C) revealed a marked increase in closure rates across all groups by Day 7. Wounds treated with PLLA/SSP-H were 29% smaller compared to the control group and 14% smaller compared to the PLLA treatment group. These results demonstrate the great potential of PLLA/SSP as an active wound dressing in promoting wound healing.

### Histological analysis of regenerative skin tissue

**Figure 7.**
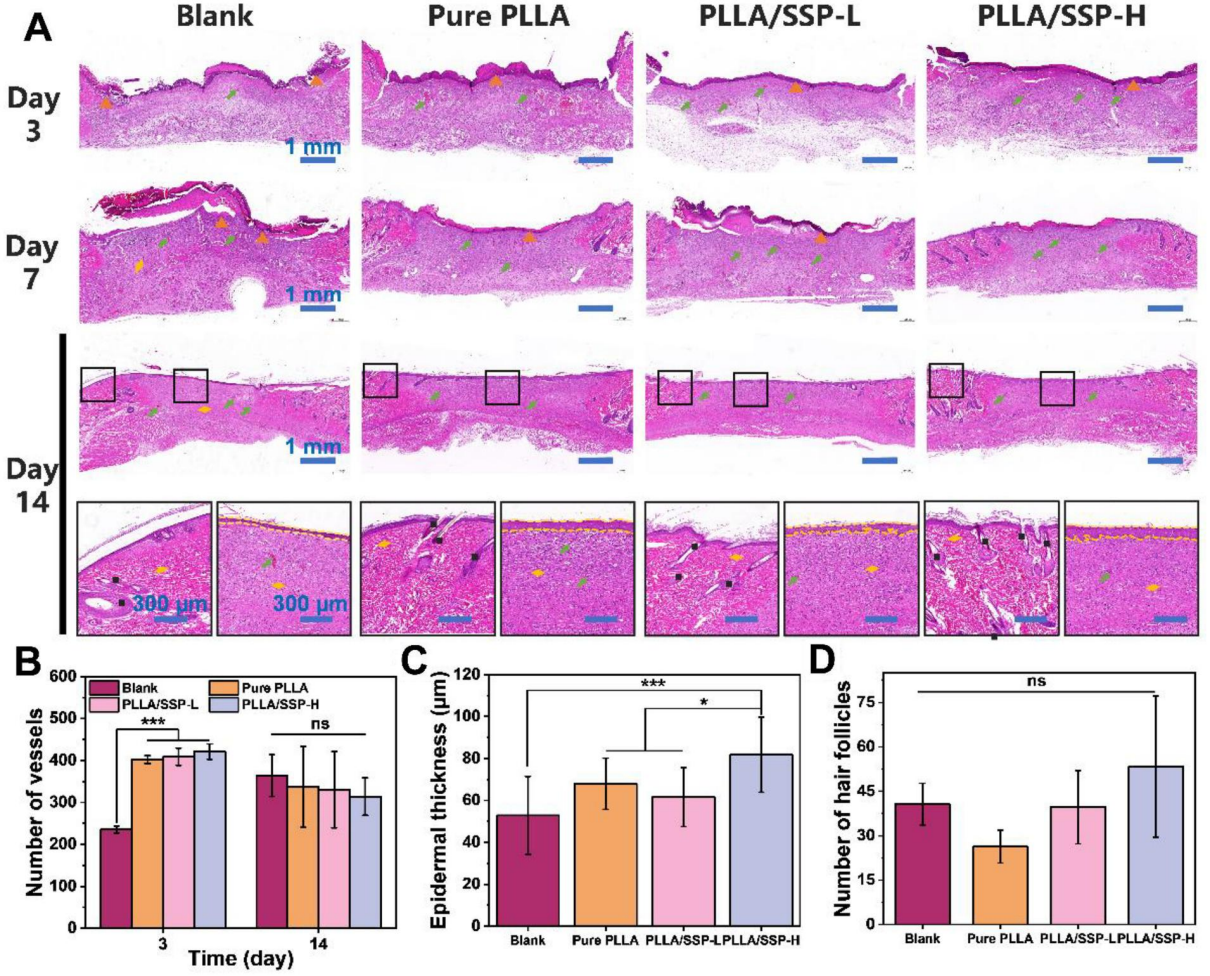
Hematoxylin-eosin (H&E) stain of wound tissues that were treated with different nanofibers. (**A**) Representative H&E staining images of wound tissue on Day 3, 7 and 14 (top three rows, scale bar = 1 mm) and magnified views of the mature area at the wound edge (left) and the immature central region (right) on Day 14 (bottom row, scale bar = 300 μm). The arrows: neovascularization; the triangles: inflammatory areas; the diamonds: fibroblasts; and the squares: newly formed hair follicles. Quantitative results of number of vessels (**B**), epidermal thickness (**C**) and number of hair follicles (**D**) are presented. * and *** denote statistically significant differences between different groups with *P* < 0.05 and *P* < 0.001, respectively. The ‘ns’ denotes *P* > 0.05, representing no significant difference.

**Figure 8.**
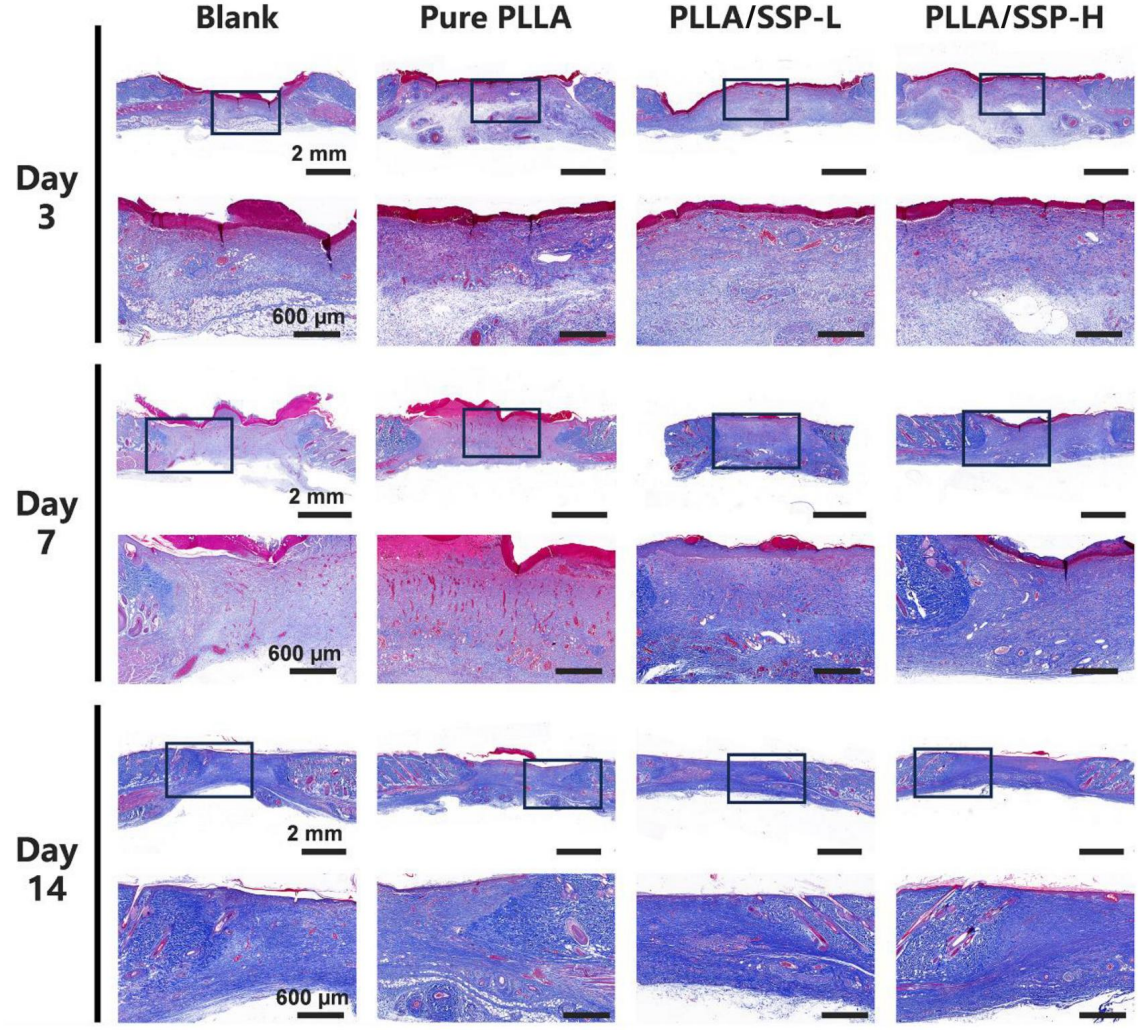
Masson’s trichrome staining of wound tissues treated with different nanofibers (panoramic and magnified images with scale bars of 2 mm and 600 µm, respectively).

To further investigate the effects of PLLA/SSP dressing on skin tissue regeneration, histological analyses were performed. Tissues harvested on Day 3, 7 and 14 were subjected to hematoxylin and eosin (H&E) staining (Figure 7) and Masson’s trichrome staining (Figure 8). During the healing phases at Day 3 and 7, all experimental groups showed consistent epidermal regeneration, granulation tissue formation, angiogenesis and collagen fiber deposition. Notably, the PLLA/SSP-H group exhibited the highest level of angiogenesis, the lowest degree of inflammatory infiltration and the smallest defect size. On Day 3, the PLLA/SSP-H group also had the most regenerated blood vessels (Figure 7B), corroborating results from the tube formation assay and highlighting its ability to promote angiogenesis. The numerous microvessels provided sufficient oxygen and nutrients, laying the foundation for the formation and repair of new tissue. By Day 14, representing the maturation phase of healing, the wound healing status was clearly evident. Observations of the wound margins and center showed that the PLLA/SSP-H group exhibited the highest degree of re-epithelialization, as evidenced by both the thickness of the new epidermis and the undulation of the basal layer (Figure 7C). Additionally, the PLLA/SSP-H group displayed abundant hair follicles (Figure 7D) and fewer microvessels, indicative of a higher degree of vascular remodeling (Figure 7B), reflecting the superior quality of wound healing promoted by PLLA/SSP-H treatment. Masson’s trichrome staining revealed enhanced collagen deposition and improved collagen alignment in wounds treated with PLLA/SSP-H, resulting in well-formed, orderly arranged collagen bundles (Figure 8). Moreover, subcutaneous implantation of the SSP film and tissue staining were performed (Supplementary Figure S7). By Day 14, minimal inflammatory cell infiltration was observed around the SSP film, with nearly no foreign body reaction. This observation underscores the excellent biocompatibility of SSP and corroborates the reduced inflammatory infiltration associated with PLLA/SSP in wound healing.

## Conclusion

In this study, we developed a pro-angiogenic electrospun wound dressing by embedding natural SSP into PLLA nanofibers. The incorporation of SSP optimized both the microstructure and bioactivity of the PLLA fibers. For example, the increase in SSP content led to significant modulation of fiber diameter, resulting in nanofibers as small as 417 nm in average diameter. The doping of SSP facilitated blood coagulation during hemostasis and, more importantly, enhanced angiogenesis. In tube formation assays, a weight ratio of 3:100 (SSP : PLLA) led to a 16.3% increase in vessel formation and a 118.6% increase in vascular branching. This angiogenic enhancement translated into significantly improved wound healing, with the PLLA/SSP-H group demonstrating a 29% faster closure rate compared to the control group. Additionally, the PLLA/SSP-H group exhibited superior re-epithelialization, collagen deposition and hair follicle regeneration. Overall, the SSP-embedded wound dressing holds great promise for clinical applications in wound care, particularly for chronic and ischemic wounds.

## Supplementary Material

rbaf078_Supplementary_Data
