# Supplementary material for: A pro-angiogenic wound dressing embedded with natural spider silk protein
Source: Regen Biomater. 2025 Jul 29;12:rbaf078. doi: 10.1093/rb/rbaf078 (PMC12582389; doi:10.1093/rb/rbaf078)
Supplement: rbaf078_Supplementary_Data [file rbaf078_supplementary_data.zip › Supplementary Files.pdf]

A Pro-Angiogenic Wound Dressing Embedded with Natural Spider Silk Protein

Sai Yan[1], Zhou Zhang[1], Yuheng Song[1], Juan Zhao[2], Hanrui Wang[2], Xiang Fei[1*], Ran Cao[1,3*], Meifang Zhu[1*]

1. State Key Laboratory of Advanced Fiber Materials, College of Materials Science and Engineering, Donghua University, Shanghai 201620, China
2. Hainan Spider King Biotechnology Co., Ltd., Haikou 570125, China
3. Shanghai Engineering Research Center of Nano-Biomaterials and Regenerative Medicine, Donghua University, Shanghai 201620, China

Corresponding authors: Ran Cao (rancao@dhu.edu.cn; caoranaw@sina.com.); Xiang Fei (xiangfei@dhu.edu.cn); Meifang Zhu (zmf@dhu.edu.cn).

Gel permeation chromatography (GPC)

GPC measurements of the polymer were performed on an Agilent 1260 system. The analyses were conducted at room temperature using hexafluoroisopropanol (HFIP) as the mobile phase at a flow rate of 1.0 mL/min. The injection volume was 50 μL, with a total elution time of 30 min for the chromatograms.

Water vapor transmission Rate (WVTR)

Membranes with a diameter of 10 mm were prepared using a hole punch. A 2 mL centrifuge tube was filled with 0.5 mL of deionized water. The membrane was placed over the opening of the tube (with an opening area denoted as A), completely sealing it. The initial weight (W_0) was recorded, and then the setup was placed in a constant temperature and humidity chamber (35% humidity, 37°C). The weight at different time intervals (W_t) was measured. The water vapor transmission rate (%) was calculated using the formula: WVTR (%) = (W_0 - W_t) / A × 100%.

Zeta potential measurement

The zeta potential of the electrospun membranes was measured by Electrokinetic analyzer for solid surface analysis (SurPASS 3).

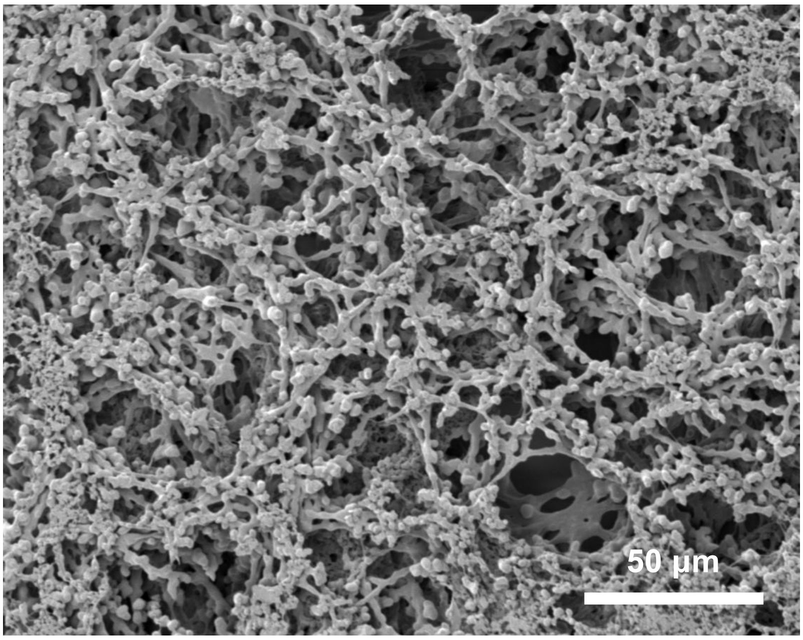

Fig. S1 SEM image of the purified SSP.

Table S1 GPC data relating to the molecular weight of SSP

Mp (g/mol)	Mn (g/mol)	Mw (g/mol)	Mz (g/mol)	Mz+1 (g/mol)	Mv (g/mol)	PD
87579	106983	127155	155446	188851	123513	1.189

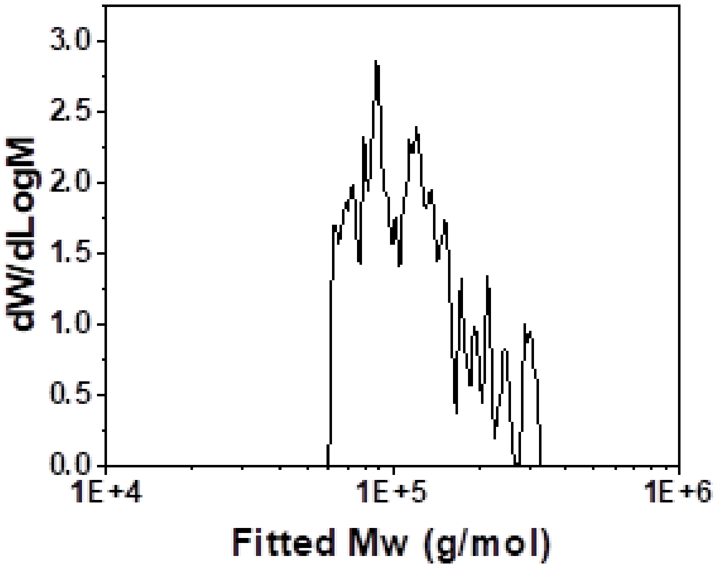

Fig. S2 GPC Chromatograms of SSP.

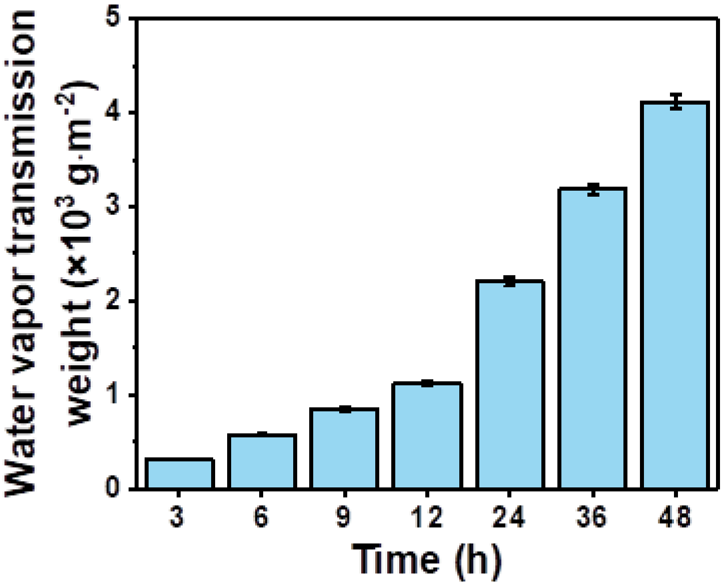

Fig. S3 Water vapor transmission rate of electrospun PLLA-SSP-H at 3, 6, 9, 12, 24, 36 and 48 h.

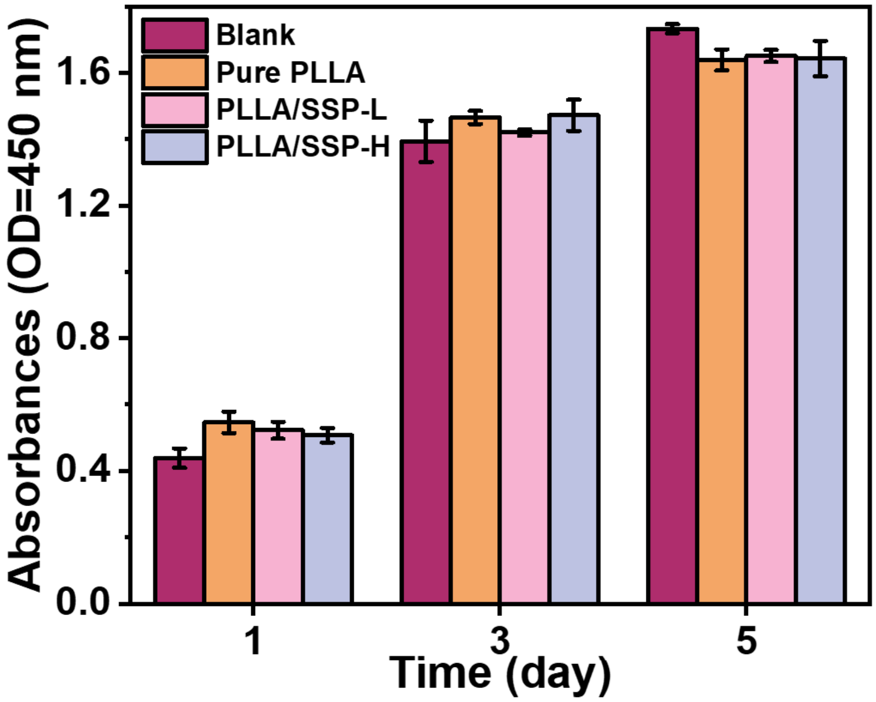

Fig. S4 Absorbances of L929 cells stained with CCK-8 for different sample treatments.

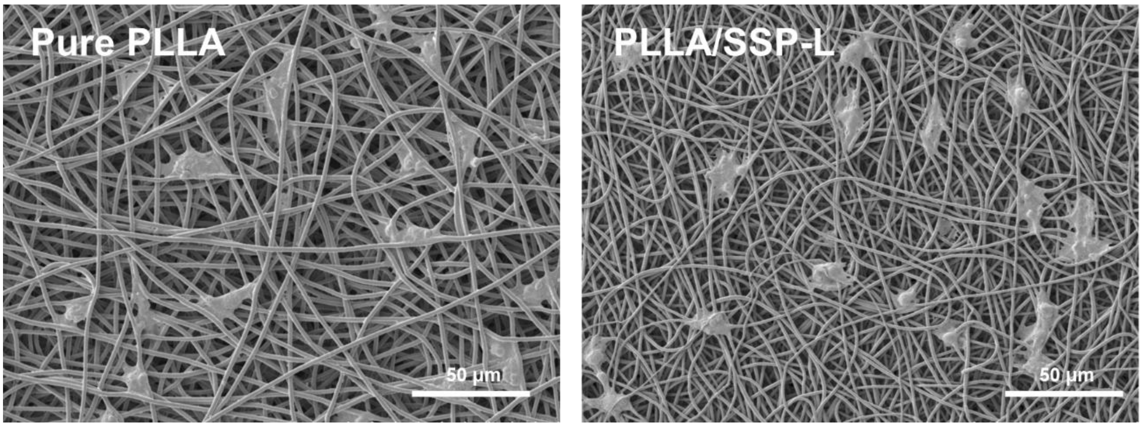

Fig. S5 SEM images of L929 cells adhered to pure PLLA and PLLA/SSP-L for 3 days.

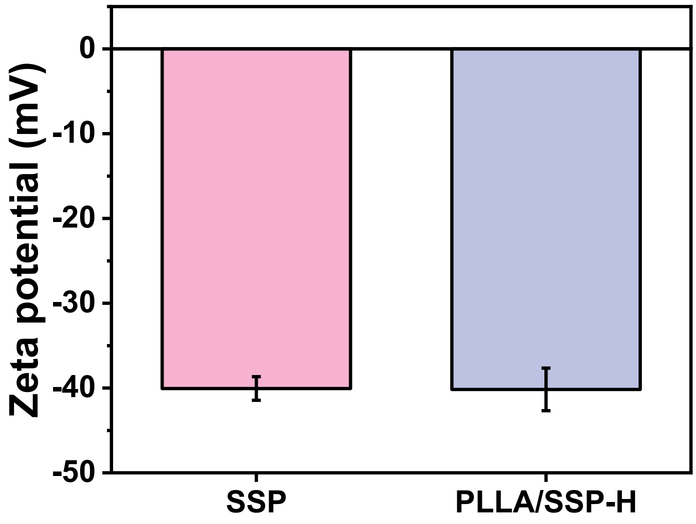

Fig. S6 Zeta potential of SSP and PLLA/SSP-H.

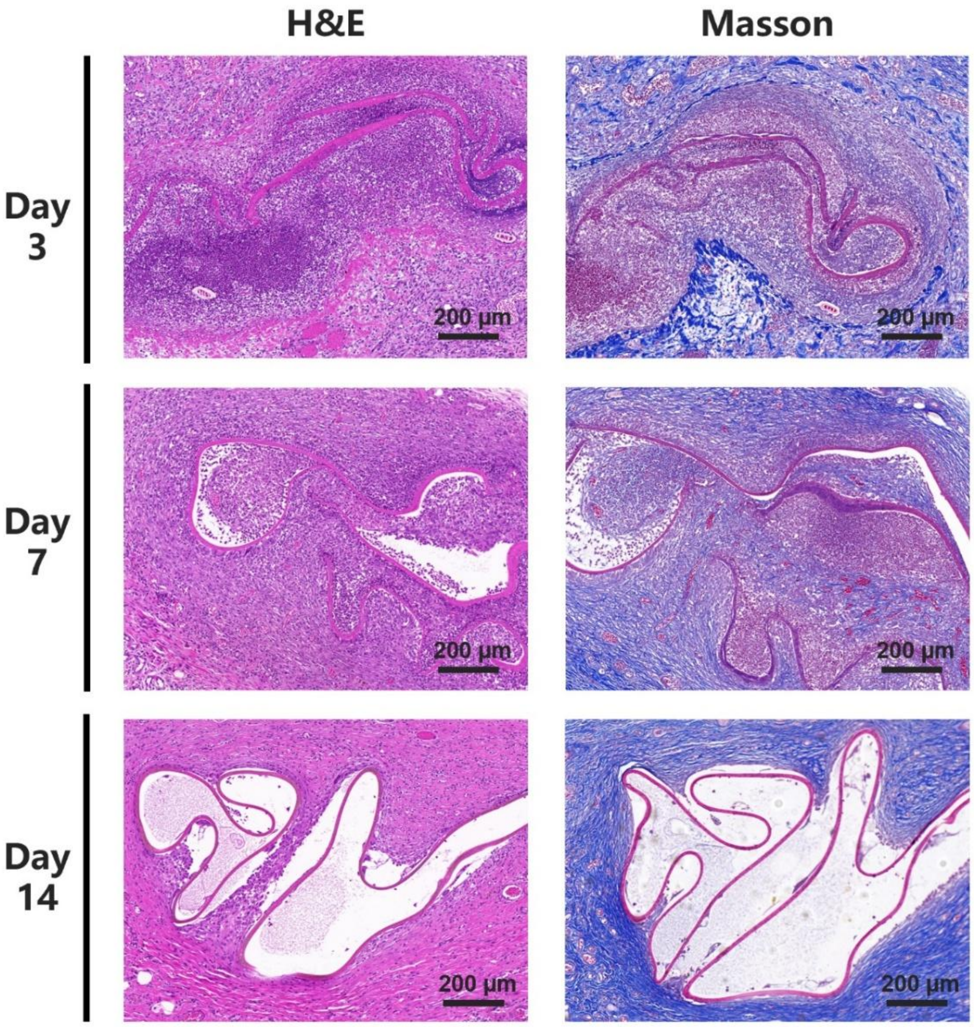

Fig. S7 Histological assessment of H&E and Masson staining of subcutaneously implanted SSP tissues on day 3, 7, and 14.
